# Case report: Rapid-onset parkinsonism after a hornet sting

**DOI:** 10.3389/fneur.2024.1365199

**Published:** 2024-04-03

**Authors:** Svetlana Tomic, Milorad Zjalic, Zvonimir Popovic, Zdravka Krivdic Dupan, Marija Heffer, Darija Snajder Mujkic, Dario Mandic, Silva Guljas, Igor N. Petrovic

**Affiliations:** ^1^Department of Neurology, Osijek University Hospital Center, Osijek, Croatia; ^2^Faculty of Medicine, Josip Juraj Strossmayer University of Osijek, Osijek, Croatia; ^3^Department of Molecular Medicine and Biotechnology, Faculty of Medicine, University of Rijeka, Rijeka, Croatia; ^4^Department of Radiology, Osijek University Hospital Center, Osijek, Croatia; ^5^Institute of Nuclear Medicine and Radiation Protection, Osijek University Hospital Center, Osijek, Croatia; ^6^Institute of Laboratory Diagnostics, Osijek University Hospital Center, Osijek, Croatia; ^7^Department of Neurology, University Clinical Center of Serbia, Belgrade, Serbia; ^8^Faculty of Medicine, University of Belgrade, Belgrade, Serbia

**Keywords:** rapid onset, parkinsonism, basal ganglia damage, sting, insect

## Abstract

Neurological manifestations with basal ganglia involvement following *Hymenoptera* stings are rare and clinically ill-defined conditions. We present a patient with acute parkinsonism non-responsive to levodopa, who developed striatal lesions after a hornet sting. We report his response to immunomodulatory treatment and subsequent clinical and brain magnetic resonance imaging (MRI) follow-up. We also searched the literature for patients with acute extrapyramidal syndromes following an insect sting. Fourteen cases have been published; 12 of them are reviewed here. The majority of cases presented with symmetric akinetic syndrome with axial rigidity and/or gait impairment. Six patients were treated with levodopa and only two of these had a modest response to therapy. Brain MRI/computed tomography scan revealed lesions of the basal ganglia, which resulted in fatal outcome in four patients, whereas only one achieved complete recovery. Clinicians should be aware of this rare but devastating cause of acute-onset parkinsonism and specific clinical presentation of this condition, and should consider prompt and prolonged immunomodulatory treatment to prevent irreversible basal ganglia damage.

## Introduction

Neurological manifestations of *Hymenoptera* stings are rare and mostly present with central or peripheral demyelination syndrome, intracranial hemorrhage and stroke (1–4). In addition, involvement of basal ganglia including pallidostriatal necrosis, coupled with different types of acute-onset parkinsonism, has been previously reported (5–17). Clinical presentation, treatment reaction and disease outcome are still unknown in this rare condition. We present a patient with acute parkinsonism non-responsive to levodopa and striatal lesions after a hornet sting, and document his response to immunomodulatory treatment with clinical and brain MRI follow-up. We also reviewed the literature for similar cases and discuss clinical presentation and prognosis of this rare condition.

## Case report

A 70-year-old, right-handed Caucasian male was referred to our department for a second opinion 2 months after he had developed acute neurological symptoms following a hornet sting. On July 22, 2020, the patient had an anaphylactic reaction with hypotension and a brief loss of consciousness soon after a hornet had stung him in the nose. He was followed for the next 24 h in his local general hospital and discharged the next day after complete recovery following antihistamine therapy with desloratadine.

Over the next 3 days, he developed a feeling of malaise and noticed that his walk had become slower and with smaller steps. Desloratadine was discontinued after 8 days, but his gait became even worse with severe hesitation at gait initiation and sudden episodes during walking when he was unable to take a step. In addition, his family members noticed that he was more taciturn and slower to respond to questions. Up until the hornet incident, he had no major health problems except for well-controlled high blood pressure treated with lisinopril.

## Diagnostic assessment

**Figure 1:**
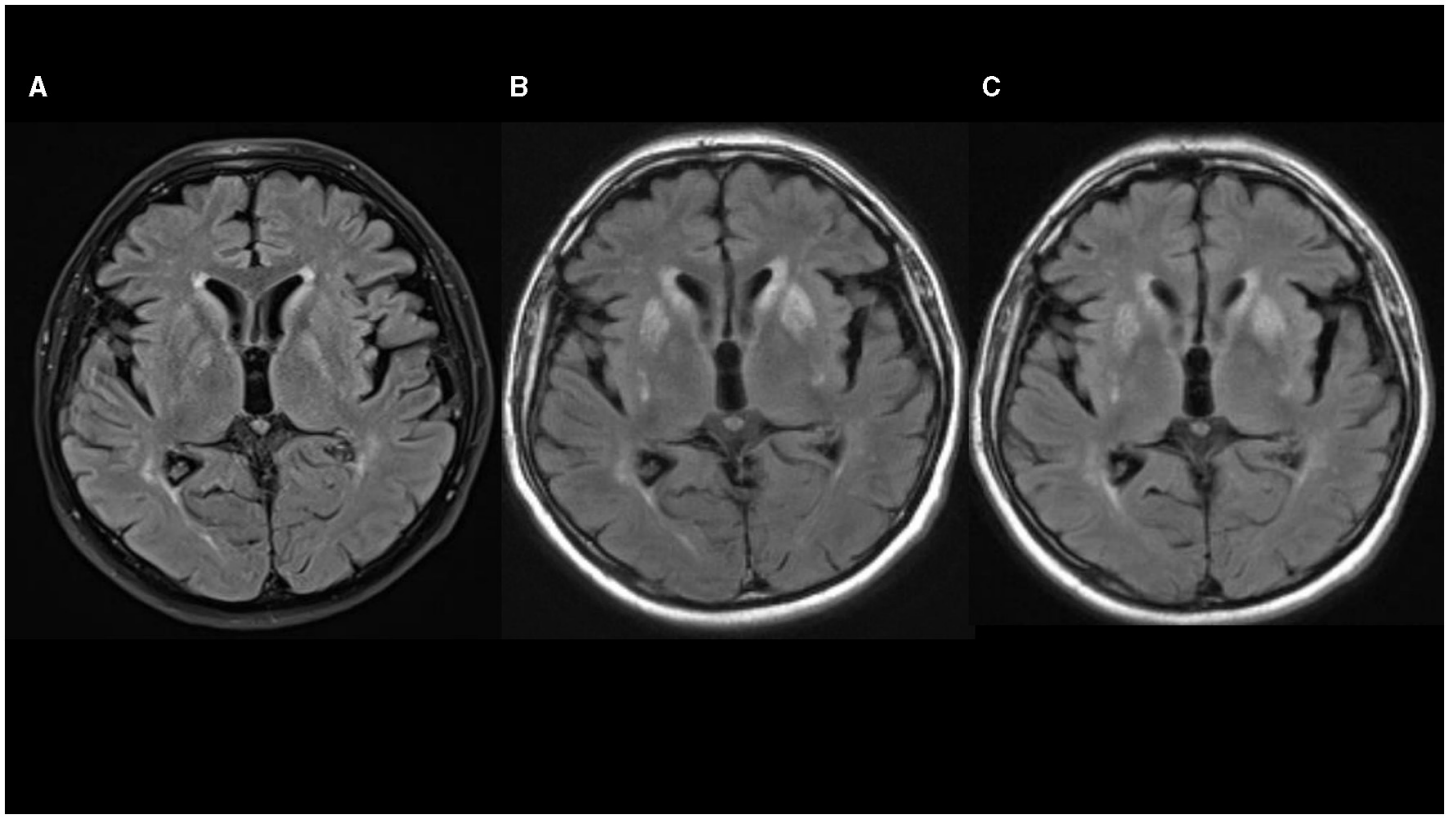
**(A–C)** Fluid-attenuated inversion recovery (FLAIR) sequences in axial plane show progression of hyperintensities in basal ganglia [**(A)** 08/2020; **(B)** 10/2020; **(C)** 02/2021].

Non-contrast axial computed tomography (CT) scan of his brain performed 2 weeks after the hornet sting did not show any signs of acute stroke, tumor, or intracranial hemorrhage. Four days later, non-contrast head MRI showed a slight bilateral striatum hyperintensity signal (HIS) on T1-weighted images, mixed bilateral striatum signal intensity on T2-weighted images with HIS on fluid-attenuated inversion recovery (FLAIR) images, without restriction of diffusion, suggesting petechial blood products corresponding to a T2^*^ susceptibility artifact on SWI sequence (Figure 1A). Levodopa/carbidopa was started and titrated slowly up to 1000 mg *per* day for the next 3 weeks, but no appreciable improvement was noted.

Two months after the hornet incident, he came to our hospital. On examination, the patient had an impassive face, speech was hypophonic and he manifested palilalia. He had symmetric rigidity of his neck and limbs, and mild symmetric bradykinesia was present in all extremities without tremor. Reflexes were brisk but his plantar responses were normal. On postural reflex testing, he recovered with 2–3 steps. The main problem was his gait with start hesitation and freezing, especially on turning or passing through the door (Supplementary Video 1_Segment 1).

His Movement Disorder Society-Unified Parkinson's Disease Rating Scale (MDS-UPDRS) III score was 35 and he was 2.5. on the Hoehn and Yahr scale. Levodopa/carbidopa treatment was slowly down-titrated and stopped. Lumbar puncture showed a small increase in protein content (0.548 g/L; reference interval 0.170–0.370 g/L) with normal cell numbers. There were no oligoclonal bands or signs of immunoglobulin intrathecal synthesis. Autoimmune antibodies (anti-NMDA-R, anti-AMPA-R1, anti-AMPA-R2, anti-GABAB-R, anti-LGI 1 and anti-CASP-R 2) from cerebrospinal fluid were normal. DaTSPECT showed normal findings. Non-contrast head MRI showed mixed striatum signal intensity on T1-weighted images with hyperintensity in ventral putamen, hyperintensity on T2-weighted and FLAIR images, predominantly in bilateral putamen and caudate nuclei with a patchy restricted diffusion-weighted image signal suggesting necrosis (Figure 1B).

**Figure 2:**
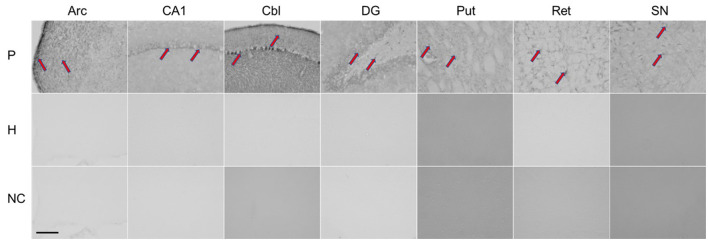
Immunohistochemical staining of rat brain slices using human serum as source of the primary antibodies. Red arrows indicate positive staining reaction in different regions of the rat brain. P, patient; H, healthy control; NC, negative controle; Arc, arcuatus; CA1, cornu ammonis 1 region of hippocampus; Cbl, cerebellum; DG, dentate gyrus of hippocampus; Put, putamen; Ret, reticular formation of pons; SN, substantia nigra. Total magnification of individual image 200x, scale 200 micrometers.

Immunohistochemistry was performed using the patient's serum to evaluate antibodies in brain tissue (for procedure, see additional material). As shown in Figure 2, rat brains reacted with the patient's serum in several regions (nucleus arcuatus, cornu ammonis 1 region of the hippocampus, cerebellum, dentate gyrus of the hippocampus, putamen, reticular formation of the pons and substantia nigra).

With intravenous corticosteroid treatment (methylprednisolone 250 mg/day for 5 days slowly tapered down over the subsequent 2 weeks), marked improvement of gait was observed (Supplementary Video 1_Segment 2). At discharge, the patient still had hesitation while turning, but freezing of gait had ceased, his MDS-UPDRS III score was 18 and he was 2 on the Hoehn and Yahr scale. Continuation of oral pronisone therapy with gradual dose tapering was recommended. Over the next 6 weeks (during which the patient stopped corticosteroid therapy on his own), gradual deterioration of gait with reappearance of freezing occurred. On that time his MDS- UPDRS III score was 30. Five courses of plasma exchange followed by rituximab were administered but without any significant gait improvement.

At that time, head MRI scan (February 28, 2021) showed mixed striatum signal intensity and hyperintensity in the ventral putamen on T1-weighted, hyperintensity on T2-weighted and FLAIR MRI images, suggesting atrophy and gliosis of the striatum, without restriction of diffusion and without contrast enhancement on T1-weighted postcontrast images (Figure 1C). Symptoms remained unchanged and no new symptoms appeared over the next 6 months of follow-up.

## Discussion

Basal ganglia necrosis associated with parkinsonism after an insect sting is a rare and ill-defined condition. In our case and the 12 previous cases from the literature (Table 1), presenting features consisted mainly of symmetric akinetic-rigid syndrome which developed within several days of the incident. Resting tremor was rare and mild, and in the majority of cases levodopa response was poor. Instead, the clinical picture consisted of gait impairment including freezing of gait, postural instabilities, and speech disturbances (including palilalia), all of which have been previously recognized as signs of pallidal damage (18). In addition, profound micrographia and akinetic mutism, which were presenting symptoms in one patient each, further support pallidal involvement (19). Interestingly, pyramidal signs including clonus and Babinski sign were noted in five out of eight patients (for the remaining cases, data are lacking). The combination of pyramidal signs coupled with specific, predominantly axial parkinsonism and gait disturbances after an insect sting more closely resemble distinctive, mainly genetically-determined pallido-pyramidal syndrome than Parkinson's disease (20).

**Table 1:** Clinical characteristics, MRI findings, treatment response and outcomes of patients who developed parkinsonism following insect stings.

**References**	**Species**	**Age (yr)**	**Anaphylaxis and latency to symptom onset**	**Extrapyramidal symptoms**	**Pyramidal symptoms**	**Brain MRI/CT scan**	**DaTSPECT**	**Pathology**	**L-dopa treatment**	**Immunoth response**	**Disease course and outcome**
Tomic et al. (this case report)	Hornet	70	Anaphylaxis 3 days	Freezing of gait; bilateral symmetrical parkinsonism	No signs of lesions	MRI 1—slight bilateral striatum HIS on T1w images, mixed bilateral striatum signal intensity on T2w images with HIS on FLAIR images, without the restriction of diffusion. MRI 3—mixed striatum signal intensity and HIS in ventral putamen on T1w, HIS on T2w and FLAIR images	Normal	Not done	No response to 1,000 mg/day	corticosteroide; PE; rituximab	Partial improvement after the first 2 weeks of treatment with methylprednisolone; after discontinuation of corticosteroides returning of symptoms without further improvement
Leopold et al. (6)	Wasp	49	No anaphylaxis A few hours	Mild resting right hand tremor; bilateral symmetric parkinsonism; postural instability	Symmetrical exaggerated muscle stretch reflexes	MRI 1—bilateral HIS signal in the globus pallidum on T2w images MRI 2—marked destruction of the striatum and pallidum bilaterally	Marked decrease in metabolic activity of the basal ganglia with sparing of the thalamus	Not done	No response to 900 mg/day	PE; IvIg; azathioprine	Stable for 6 months; followed by rapid progression Partial but significant improvement after immunomodulatory therapy
Agarwal et al. (13)	Honeybee	No data	No data 3 days	Symmetric parkinsonism; short shuffling gait	No signs of lesions	MRI—diffuse HIS on T2w and FLAIR images in bilateral caudate and lentiform nuclei; diffuse effacement of the sulci on the right frontal lobe	No data	Not done	Levodopa/carbidopa	Not given	Symptomatic improvement within 4 weeks; after 3 months parkinsonism had improved
Mittal et al. (5)	Honeybee	46	Anaphylaxis 2 days	Symmetric parkinsonism; hypokinetic dysarthria.	No signs of lesions	No data	No data	Not done	Levodopa/carbidopa	Not given	Symptomatic improvement of speech over the next 2 months; no data for outcome
van Agt et al. (7)	Wasp	52	Anaphylaxis No data	Bradykinesia; rigidity	No signs of lesions	MRI—symmetrical bilateral caudate nuclei and putaminal HIS signal in T1w, T2w and FLAIR images. Iron deposition in the right external capsule in T2w images.	No data	Not done	Levodopa/carbidopa	Not given	Mild improvement with levodopa after 6 months
Gallego et al. (8)	Wasp	72	No anaphylaxis 1 h	Rigidity	Right hemiparesis bilateral Babinski sign; symmetrically brisk tendon jerks	MRI not done; CT scan—low density of both lenticular nuclei, most pronounced in the left globus pallidus	Not done	Bilateral cavitations of the globus pallidus and softening of the putamen and caudate nuclei in subcortical white matter pallor of the myelin	Not given	Not given	Death (72 h after the sting)
Castaigne et al. (10)	Wasp	No data	No data	No data	No data	Not done	Not done	Severe necrotic lesions in the putamen and less severe lesions in the caudate, thalamus, and red nucleus	Not given	Not given	Coma; death after 55 days
Bogolepov et al. (11)	Wasp	51	Anaphylaxis 1 h	Parkinsonism	No data	Not done	Not done	Bilateral pallidal necrosis with less profound lesions in the substantia nigra	Not given	Not given.	Death after 14 days
Laplane et al. (9)	Wasp	No data	No anaphylaxis No data	Chorea; buccofacial dyskinesias; compulsive movements	No data	MRI not done; CT scan –bilateral hypodense pallidostriatal lesions	Not done	Not done	Not given	Not given	Gait disorder and myoclonus lasted for several months with slow recovery; compulsive obsessive behavior developed several years later
Gale (12)	No data	36	Anaphylaxis 1 day	Dystonia; symmetric parkinsonism	Brisk tendon reflexes	MRI not done; CT scan—low attenuation in both posterior parietal regions	Not done	Not done	No response to levodopa	Not given	Partial improvement
Gale (12)	Wasp	38	Anaphylaxis A few hours	Akinetic mutism; plastic hypertonicity in the limbs; catatonic posturing	Tendon reflexes symmetrically brisk; Babinski sign bilaterally; clonus	Not done	Not done	Not done	Not given	Not given	Some improvement; regained speech; died 4 months later from pulmonary embolism
Sehgal et al. (16)	Wasp	No data	No data 2 days	Akinetic-rigid parkinsonism	No data	No data	No data	No data	No data	No data	No data
Kumawat et al. (17)	Honeybee	40	No anaphylaxis 3 days	Symmetric akinetic-rigid parkinsonism; extrapyramidal dysarthria; dystonic posture on all four limbs	No signs of lesions	MRI - in T2w and FLAIR images HIS signals in bilateral basal ganglia and left centrum semioval	Not done	Not done	Not given	Corticosteroide intravenously for 5 days	All symptoms and signs resolved promptly; no data on follow-up

^*^Minault et al., Nouv Presse Med 1981, article in French; Mavra et al., Srp Arh Celok Lek. 1983—not available.

CT, computed tomography; HIS, hyperintensive signal; IvIg, intravenous immunoglobulins; MRI, magnetic resonance imaging; PE, plasma exchange; T1w, T1-weighted; T2w, T2-weighted.

MRI was performed in six out of 12 patients, and HIS on T2-weighted and FLAIR images in the region of basal ganglia (striatum, mainly pallidum), were noted in all of them. In three additional cases, CT scans also demonstrated mainly symmetric hypodensity in lenticular nuclei. In our patient, non-contrast brain CT scan and brain MRI performed 2–3 weeks after the hornet sting showed bilateral petechial blood products in the striatum. Non-contrast brain MRI performed 3 months after hornet sting showed signs of bilateral striatal necrosis, and follow-up brain MRI performed after 7 months showed atrophy and gliosis of the striatum. Combining those data with normal DaTSPECT in our case suggest a postsynaptic cause of parkinsonism predominantly due to striatal damage with sparing of the nigrostriatal dopamine bundle.

The etiology of basal ganglia damage in this rare condition is unknown. Hypoxic-ischaemic mechanism due to an exaggerated anaphylaxis, associated hypotension, and eventual prolonged cerebral hypoxia is one possible mechanism. This sequence of events might explain cases with severe hypotension, prolonged loss of consciousness and respiratory failure. Nevertheless, in case presented by Gallego et al. patient presented with stupor 1 h after incident, soon progressed to coma but without significant cardiovascular, electrolytic or respiratory failure (8). In addition, authors stated that histological pattern on necropsy did not suggest a hypoxic-ischaemic nature of basal ganglia damage. In case we presented, as well as in other cases collected here the anaphylactic reaction led to hypotension without or only with short-term loss of consciousness that did not require cardiopulmonary resuscitation, and there was no need for respiratory support suggesting the hypoxic-ischemic mechanism of brain damage unlikely (6, 17).

Given the acute onset of parkinsonism and neuroimaging findings, an immune-mediated early hypersensitivity reaction is most likely. In support of this are evidence for autoimmunity obtained from immunohistochemistry and the excellent treatment response observed in one patient who received corticosteroids in the acute phase, as well as the beneficial but limited clinical response to corticosteroid treatment observed in our case and those reported by Leopold et al. (6). In the latter two cases, immunomodulatory treatment was applied with significant delay. Although we found that antibody reacted with different brain regions (nucleus arcuatus, cornu ammonis 1 region of hippocampus, cerebellum, dentate gyrus of hippocampus, putamen, reticular formation of pons and substantia nigra), only the striatum was damaged by necrosis. Susceptibility of the basal ganglia to toxic and hypoxic lesions has already been described in human and animal studies, and mitochondrial failure with energy deprivation is considered as the main underlying mechanism (21).

## Patient perspective

In conclusion, clinicians should be aware of this rare but devastating cause of acute-onset parkinsonism, it‘s specific clinical presentation and clinical course. Although limited, current knowledge suggest that prompt and prolonged immunomodulatory therapy could be a rational therapeutic choice in attempt to prevent irreversible basal ganglia damage.

## Data availability statement

The raw data supporting the conclusions of this article will be made available by the authors, without undue reservation.

## Ethics statement

Ethical review and approval was not required for the study on human participants in accordance with the local legislation and institutional requirements. Written informed consent from the patients/participants or patients/participants' legal guardian/next of kin was not required to participate in this study in accordance with the national legislation and the institutional requirements. Written informed consent was obtained from the individual(s) for the publication of any potentially identifiable images or data included in this article.

## Author contributions

ST: Conceptualization, Funding acquisition, Investigation, Writing—original draft, Writing—review & editing. MZ: Investigation, Writing—original draft, Writing—review & editing. ZP: Investigation, Writing—original draft, Writing—review & editing. ZK: Data curation, Investigation, Writing—original draft, Writing—review & editing. MH: Data curation, Writing—original draft, Writing—review & editing. DS: Data curation, Investigation, Writing—original draft, Writing—review & editing. DM: Data curation, Investigation, Writing—original draft, Writing—review & editing. SG: Data curation, Investigation, Writing—original draft, Writing—review & editing. IP: Conceptualization, Formal analysis, Methodology, Project administration, Resources, Validation, Writing—original draft, Writing—review & editing.
